# Groundtruthing Next-Gen Sequencing for Microbial Ecology–Biases and Errors in Community Structure Estimates from PCR Amplicon Pyrosequencing

**DOI:** 10.1371/journal.pone.0044224

**Published:** 2012-09-06

**Authors:** Charles K. Lee, Craig W. Herbold, Shawn W. Polson, K. Eric Wommack, Shannon J. Williamson, Ian R. McDonald, S. Craig Cary

**Affiliations:** 1 Department of Biological Sciences, University of Waikato, Hamilton, New Zealand; 2 Center for Bioinformatics and Computational Biology, Delaware Biotechnology Institute, University of Delaware, Newark, Delaware, United States of America; 3 Department of Computer and Information Sciences, University of Delaware, Newark, Delaware, United States of America; 4 Department of Biological Sciences, University of Delaware, Newark, Delaware, United States of America; 5 Department of Plant and Soil Sciences, University of Delaware, Newark, Delaware, United States of America; 6 College of Earth, Ocean, and Environment, University of Delaware, Lewes, Delaware, United States of America; 7 J. Craig Venter Institute, San Diego, California, United States of America; Argonne National Laboratory, United States of America

## Abstract

Analysis of microbial communities by high-throughput pyrosequencing of SSU rRNA gene PCR amplicons has transformed microbial ecology research and led to the observation that many communities contain a diverse assortment of rare taxa–a phenomenon termed the *Rare Biosphere*. Multiple studies have investigated the effect of pyrosequencing read quality on operational taxonomic unit (OTU) richness for contrived communities, yet there is limited information on the fidelity of community structure estimates obtained through this approach. Given that PCR biases are widely recognized, and further unknown biases may arise from the sequencing process itself, *a priori* assumptions about the neutrality of the data generation process are at best unvalidated. Furthermore, post-sequencing quality control algorithms have not been explicitly evaluated for the accuracy of recovered representative sequences and its impact on downstream analyses, reducing useful discussion on pyrosequencing reads to their diversity and abundances. Here we report on community structures and sequences recovered for *in vitro*-simulated communities consisting of twenty 16S rRNA gene clones tiered at known proportions. PCR amplicon libraries of the V3–V4 and V6 hypervariable regions from the *in vitro*-simulated communities were sequenced using the Roche 454 GS FLX Titanium platform. Commonly used quality control protocols resulted in the formation of OTUs with >1% abundance composed entirely of erroneous sequences, while over-aggressive clustering approaches obfuscated real, expected OTUs. The pyrosequencing process itself did not appear to impose significant biases on overall community structure estimates, although the detection limit for rare taxa may be affected by PCR amplicon size and quality control approach employed. Meanwhile, PCR biases associated with the initial amplicon generation may impose greater distortions in the observed community structure.

## Introduction

High-throughput pyrosequencing of PCR amplicons has emerged as a valuable technique in microbial ecology and revealed, in unprecedented detail, the microbial diversities found in various marine and terrestrial environments [1]–[9] and the human microbiome [10]–[13]. The power of this approach lies in the read depth achieved, where tens to hundreds of thousands of individual sequencing reads are simultaneously generated and used to estimate the composition and abundance of microbial operational taxonomic units (OTUs) in a given community. However, this high read depth comes at a cost of relatively high error rates for individual reads obtained using commonly employed sequencing technology (i.e., Roche 454 GS FLX with Titanium chemistry, 454-Ti) [14]. In the context of genomic (re-) sequencing, low consensus error rates are achieved through sequence assembly; however, for PCR amplicons, redundancy is indistinguishable from abundance, and the high error rates associated with individual reads therefore contribute to over-estimation of diversity since erroneous reads manifest themselves as less abundant but closely related OTUs [15].

A number of attempts have been made to assess and address the impact of 454 single read errors on the estimation of community richness. These efforts have primarily addressed the accuracy of OTU diversity estimates, with special attention paid to enumeration of OTUs within the *Rare Biosphere* [6], [15]–[17]. One consistent finding has been that standard techniques for processing amplicon pyrosequencing data can result in the detection of several hundred “false” OTUs, mostly at low abundance, even from a single test organism [15]. Those findings have raised concerns that species abundance can be overestimated for amplicon pyrosequencing data. Subsequently, more stringent approaches have been developed that allow the abundances of error-containing reads to be counted toward those of the more abundant, supposedly error-free, reads from which they arose [16]–[21].

Computational strategies employed by these newly developed “de-noising” methods fall into three categories: 1) identity-based clustering, where de-noising is achieved by aligning and clustering nucleotide sequences (e.g., single-linkage pre-clustering, SLP [17]; CD-HIT-OTU, http://weizhong-lab.ucsd.edu/cd-hit-otu; and “otupipe”, http://drive5.com/otupipe/); 2) non-alignment clustering, which utilizes K-mer clustering rather than alignment-based distance calculations to de-noise reads [22] or even directly assign reads to OTUs [20]; 3) flowgram-based clustering, where information obtained by clustering pyrosequencing flowgrams is incorporated into the de-noising pipeline [18], [19], [21], [23]. All these methods also use quality filters perceived to be correlated with low read accuracy, such as abnormal read length, mismatch to barcode and/or PCR primer, and low quality score. To examine and compare the performance of these different approaches in accurately recovering community structures, we chose three published methods, SLP [17], PyroTagger [20], and AmplionNoise [19], to represent the three categories, respectively.

All de-noising pipelines assign the abundance of a “true” amplicon sequence as the sum of its own abundance and those of “noise” reads that arose from it, removing “noise” reads from the dataset in the process. However, different strategies are employed by each de-noising pipeline to determine the sequence identity of the “true” read (i.e., picking the representative sequence of each OTU). Ultimately, the fidelity of representative sequences is important for accurate taxonomic assignment and phylogenetic analysis. Moreover, over-aggressive removal of noise through clustering inevitably leads to incorrect clustering of genuine but closely related sequences that may correspond to highly distinct ecotypes [24].

A wide array of factors affects the determination of microbial community structure from 16S rRNA gene amplicons. PCR amplicon size has been suggested to impact observable diversity [25], ostensibly due to lower amplification/cloning efficiency for longer amplicons; although PCR amplicon size and primer choice are inevitably linked, and their effects are difficult to separate [26], [27]. Additional PCR biases, including primer mismatch [28], [29], differential amplification efficiency [30], [31], and differential annealing efficiency [29], can also affect observed diversity and structure. These issues, when combined with the high error rates discussed above, can distort estimates of community taxonomic richness and abundance.

The de-noising strategies outlined above have not been examined in regards to sensitivity for genuinely rare taxa or accuracy of estimated community structure. For comparative studies in particular, it is essential that the recovered read frequencies can be reliably interpreted as evidence of population abundances. Furthermore, ensuring that rare reads truly indicate rare taxa is important since they constitute the philosophical basis of the modern *Rare Biosphere* concept [6]. Therefore, the potential influence of the post-PCR pyrosequencing workflow on observed microbial community structure and diversity remains under-examined. A thorough investigation of this topic requires *a priori* knowledge of community composition and structure.

In this study, we utilized six different *in vitro*-simulated communities (*iv*-SCs) of 16S rRNA gene PCR amplicons to characterize biases associated with microbial community structure reconstruction using pyrosequencing data. To achieve this, potential skews in observed community structure, the practical detection limit for rare taxa, and the effects of PCR bias in the initial PCR step were all examined and assessed for their implications on the application of this technique for microbial ecology research.

## Results

### Community Diversity and Structure from PCR-Neutral Communities

**Figure 1:**
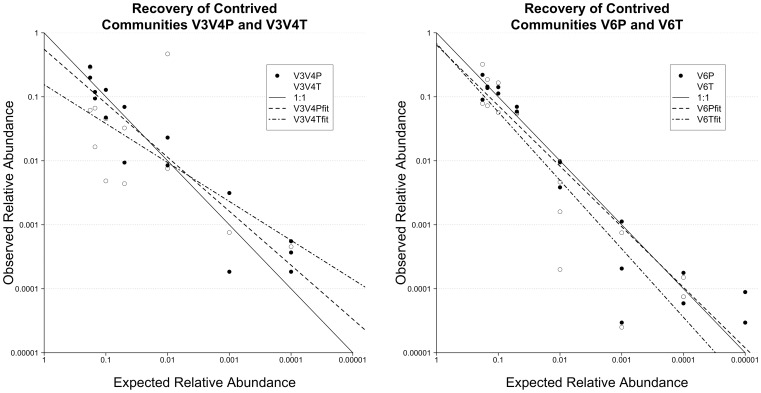
The relative abundances of recovered reads in V3V4P and V3V4T *iv*-SCs (**Figure 1A**) and V6P and V6T *iv*-SCs (**Figure 1B**) are plotted against their respective theoretical relative abundances. The solid lines represent the ideal 1∶1 scenario (i.e., observed matching expected perfectly).

PCR-independent *in vitro-*simulated communities (*iv*-SCs) V3V4P and V6P tested the neutrality of 454-Ti pyrosequencing as they were constructed using individually generated amplicons pooled at known abundances (Table 1). Of the 20 original sequences present in each dataset, 19 (95%) were recovered for V6P (36,394 reads), but only 15 (75%) were recovered for V3V4P (9,787 reads, Table S1). The frequency of each known sequence within these *iv*-SCs was recovered based on the numbers of corresponding error-free reads (i.e., sequences generated by the 454 base-calling software with default parameters that perfectly matched known sequences) (Table S2). The sole sequence missing from V6P was clone LMMI-24 in the lowest frequency tier (0.001%). Clone sequences absent from the V3V4P *iv*-SCs included all three sequences at 0.001% frequency, one sequence at 0.1%, and one of the three sequences expected at 1%. However, the higher number of sequences recovered from V6P was likely due to its higher accurate read count. Of the sequences recovered from *iv*-SCs V3V4P and V6P, observed relative abundances were generally in agreement with expected frequencies, although deviations exceeding 10-fold did occur at low expected frequencies (Figure 1). The correlation between observed and expected frequencies was consistent for both the V3V4P and V6P (PCR-controlled) communities (Table 2), with V6P resampled to match the number of error-free reads for V3V4P.

**Table 1:** Expected relative abundances of each 16S rRNA gene-containing plasmid (E and T) or amplicon (P) in the *in vitro*-simulated communities (*iv*-SCs).

	Community		
16S rRNA gene clone	Equal (E)	Tiered (T)	Tiered PCR Product (P)
4–3Okaro10‡	0.05	0.18	0.18
SC8-3†	0.05	0.18	0.18
SC7-1†	0.05	0.15	0.15
LMM1-5‡	0.05	0.15	0.15
SC1-5†	0.05	0.1	0.1
3-9‡	0.05	0.1	0.1
23-7‡	0.05	0.05	0.05
30-1‡	0.05	0.05	0.05
19-3†	0.05	0.01	0.01
16-1†	0.05	0.01	0.01
1216C**	0.05	0.01	0.01
SC5-2†	0.05	0.001	0.001
29-2†	0.05	0.001	0.001
Forsyth-N6†	0.05	0.001	0.001
Waahi-22‡	0.05	0.0001	0.0001
SC4-1‡	0.05	0.0001	0.0001
3-1†	0.05	0.0001	0.0001
6-1†	0.05	0.00001	0.00001
EF222209‡	0.05	0.00001	0.00001
LMM1-24‡	0.05	0.00001	0.00001

† Rueckert et al. 2007.

‡ Rueckert Personal Communication.

** Banks et al. 2009.

**Table 2:** Spearman rank (ρ) and log-log transformed Pearson (*r*) correlation coefficients of error-free sequences with their respective theoretical frequencies.

Community		Spearman ρ	Pearson *r*
V3V4	V3V4P	0.941	0.943
	V3V4T	0.596	0.669
V6	V6P	0.928 {0.899, 0.950}	0.961 {0.923, 0.986}
	V6T	0.923 {0.887, 0.952}	0.911 {0.855, 0.967}

The pools of error-free sequences for V6P and V6T (33,804 and 39,978 reads respectively) were resampled 10,000 times with replacement to match the numbers of V3V4P and V3V4T error-free sequences (5,424 and 6,607 reads respectively). The correlation coefficients for each bootstrap were calculated and presented as means and 95% confidence intervals. The bootstrapping *p* values (testing the V6x correlation coefficients as higher than the V3V4x equivalents) were 0.814 (ρ) and 0.152 (*r*) for resampled V6P vs. V3V4P and <0.001 (ρ and *r*) for resampled V6T vs. V3V4T.

### Effects of PCR Biases

**Figure 2:**
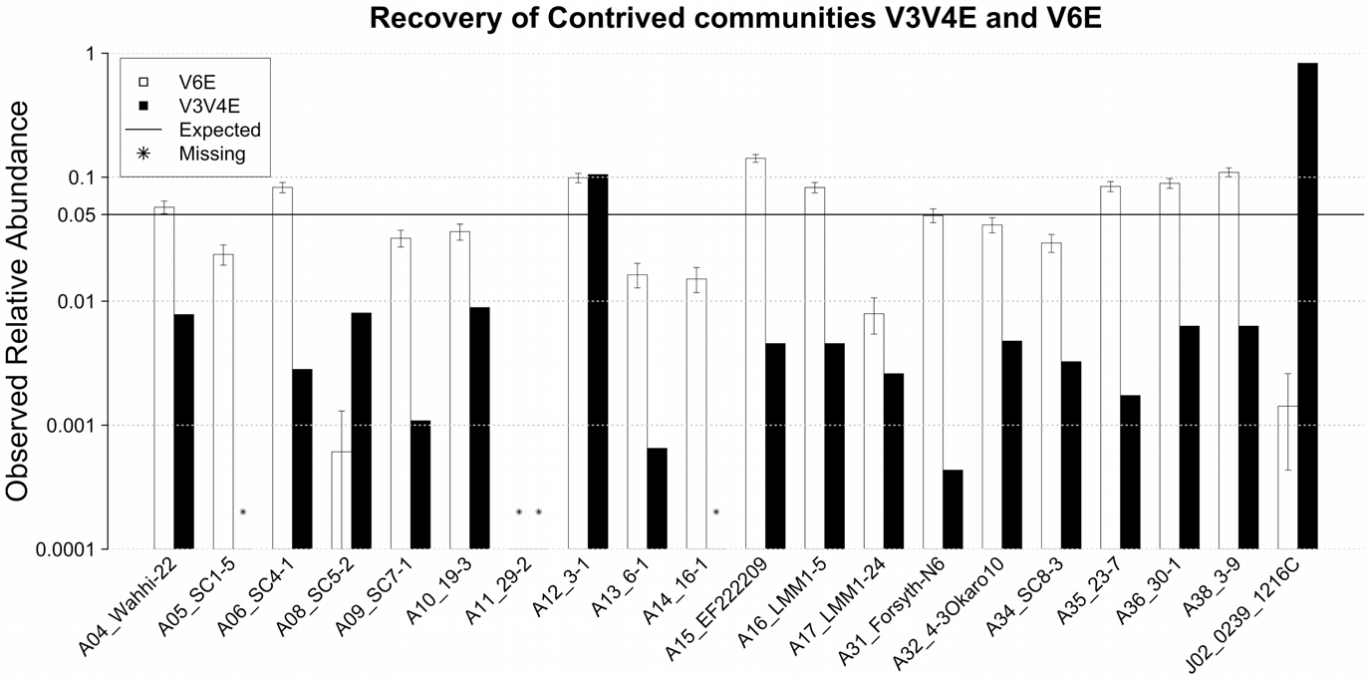
The observed relative abundances of all error-free sequence in the equal-abundance *iv*-SCs (V3V4E: red; V6E: blue). The pool of error-free sequences for V6E (14,761 reads) was resampled 10,000 times with replacement to match the number of V3V4E error-free sequences (4,609 reads) and used to calculate 95% confidence intervals for V6E.

To examine the degree to which PCR biases are sufficient to induce a non-uniform community structure into a uniform community of template DNA, an *iv*-SC set was constructed with twenty plasmids at equal abundances (Table 1). This set of *iv*-SC (V3V4E & V6E) was generated using two separate PCR assays, targeting the V3–V4 and V6 regions of 16S rRNA gene, respectively. Analysis of error-free reads from these *iv*-SCs revealed non-uniform frequency distributions of sequences (Figure 2). The observed bias does not appear to have been caused by quantification error, as the bias observed for sequence 1216C in V3V4E was so extreme that it accounted for 83% of the total dataset. Meanwhile, this clone was significantly under-represented in V6E, accounting for only 0.14% of the reads.

The influence of PCR biases on a tiered community structure was also examined using *iv*-SCs V3V4T and V6T. These amplicons were generated using twenty plasmids, pooled at tiered abundances, as PCR template (Table 1). In general, observed clone frequencies were similar to expected ones (Figure 1 & Table 2). However, as seen in *iv*-SC V3V4E, the preferential amplification of the V3–V4 region of clone 1216C was severe in V3V4T and resulted in this single sequence comprising nearly half of the total reads obtained (Figure 1A and Table S2). Overall, the observed bias in favor of a single sequence depressed the observed frequencies for other sequences and thus skewed the observed community structure. This resulted in a significantly worse correlation between the observed and expected relative abundances for the longer V3V4T amplicon community than either the V6T community (Table 2) or the PCR-controlled V3V4P community (Table 2). This difference in correlation was ameliorated by removing sequence 1216C from the analysis (Table 3).

**Table 3:** Spearman rank (ρ) and log-log transformed Pearson (*r*) correlation coefficients of relative abundances of corresponding sequences in P and T *iv*-SCs.

Comparison	N	Spearman ρ	Pearson *r*
V6P vs. V6T	16	0.973	0.953
V3V4P vs. V3V4T	12	0.748	0.794
V3V4P vs. V3V4T (Excluding 1216C)	11	0.936	0.936

### Impact of PCR Primer Mismatch on Observed Relative Abundances

The original sequences of all 20 clones (obtained using bi-directional Sanger sequencing) were examined for PCR primer mismatches that may have contributed to observed frequency biases (Table S3). Seventeen clones exactly matched both V6 primers (968F & 1046R, Table 4), with single nucleotide mismatches in the remaining three clones (Table S3). Conversely, only one clone (1216C) exactly matched both V3–V4 primers (338F & 806R, Table 4). The remaining 19 clones had mismatches of up to 5 nucleotides (Table S3). For *iv*-SCs V3V4E and V3V4T, the number of PCR primer mismatches was significantly and negatively correlated with observed/expected ratios (nonparametric Spearman correlation analysis excluding clones 19-3 and 6-1; V3V4E: *p* = 0.007; V3V4T: *p* = 0.015;). The same was true for V6E (nonparametric Mann-Whitney test; *p* = 0.0081), but not V6T (*p* = 0.0626).

**Table 4:** Unidirectional hybrid PCR primers; 454 adapter sequence in italic, MID sequence in brackets.

Primer Name	Primer Sequence
V3V4E_Forward	*CCATCTCATCCCTGCGTGTCTCCGACTCAG*[ACACGTACAG]ACTCCTACGGGAGGCAGCAG
V3V4T_Forward	*CCATCTCATCCCTGCGTGTCTCCGACTCAG*[ACACACGTCG]ACTCCTACGGGAGGCAGCAG
V3V4P_Forward	*CCATCTCATCCCTGCGTGTCTCCGACTCAG*[ACACGTCTCG]ACTCCTACGGGAGGCAGCAG
V3V4_Reverse	*CCTATCCCCTGTGTGCCTTGGCAGTCTCAG* GGACTACCAGGGTATCTAAT
V6E_Forward	*CCATCTCATCCCTGCGTGTCTCCGACTCAG*[ACAGTACGCG]AACGCGAAGAACCTTACC
V6T_Forward	*CCATCTCATCCCTGCGTGTCTCCGACTCAG*[ACACTACGAC]AACGCGAAGAACCTTACC
V6P_Forward	*CCATCTCATCCCTGCGTGTCTCCGACTCAG*[ACGACACTAG]AACGCGAAGAACCTTACC
V6_Reverse	*CCTATCCCCTGTGTGCCTTGGCAGTCTCAG*CGACAGCCATGCANCACCT

The V3V4 and V6 forward and reverse primers were based on 338F, 806R, 968F, and 1046R, respectively [27], [28], [45].

### Overview of Pyrosequencing De-noising Strategies

Three recently published algorithms for de-noising 16S rRNA gene PCR amplicon pyrosequencing libraries, SLP [17], PyroTagger [20], and AmpliconNoise [19], were examined for their ability to accurately reconstruct community structure and diversity using the PCR-independent *iv*-SCs (V3V4P & V6P). Unique reads determined by these de-noising pipelines typically represent multiple error-free and error-containing reads, the latter presumably derived from the former. Each algorithm identifies a set of presumably error-free (“true”) reads, which determine the eventual accuracy of identified OTUs.

### Community Structure Estimated in the Presence of Error-Containing Reads

To understand the behavior of each de-noising algorithm and workflow, we devised a classification scheme for OTUs comprised of read predictions. An OTU containing at least one unique read prediction (predicted by de-noising algorithm) that correctly matched one of the twenty reference clone sequences was designated a “true” OTU. An OTU containing raw reads that correctly mapped to one of the 20 reference clone sequences but whose read predictions all contained at least one error was designated as a “miscalled” OTU. An OTU comprised entirely of reads that did not match any of the 20 reference sequences was designated a “false-derived” OTU. Other designations included “near-match” OTUs, which contained sequences matching closely to a reference sequence not found in any “true” or “miscalled” OTUs; “contamination” OTUs, which generally represented *E. coli* vector contamination; and “chimeric” OTUs, which contained chimeric sequences not identified by the chimera-check algorithm. OTUs classified in this manner for *iv*-SCs V3V4P and V6P are summarized in Table 5 (details in Table S4). Since recommended clustering procedures differ for each de-noising pipeline, the 20 known sequences were clustered using each procedure in a “clustering control” (Table 5). Based on the number of OTUs obtained from the clustering controls, it was clear that the PyroTagger clustering algorithm was overly aggressive since only 12 OTUs were obtained from the 20 V3–V4 reference sequences, considerably fewer than were found by the SLP (16 OTUs) or AmpliconNoise (17 OTUs) clustering procedures (Table 5). The number of OTUs obtained from the V6 clustering controls was the same for all three pipelines.

**Table 5:** Summary statistics for the community analysis using several de-noising algorithms on the *iv*-SCs containing 20 known sequences.

		Total OTU	Chao1 Index	True OTU	Miscalled OTU	Near-Match OTU	Contami-nation OTUs	Chimeric OTU	False-Derived OTU	Missing OTU*	Clustering Control
V3V4P	SLP	14	17	8	0	0	6	0	0	7	16
	AmpliconNoise	23	51	4	7	3	5	4	0	1	17
	PyroTagger	15	15	10	0	1	2	2	0	3	12
V6P	SLP	35	48.75	13	3	2	2	6	9	0	18
	AmpliconNoise	33	49.5	14	2	1	3	5	8	0	18
	PyroTagger	22	36	13	1	1	1	3	3	3	18

For each methodology a “clustering control” was run to determine how many OTUs would be expected in the absence of errant reads.

* Missing OTU numbers exclude reference sequences that were unidentifiable in the raw dataset (5 missing in V3V4P dataset and 1 missing in V6P dataset).

The ability of de-noising algorithms to identify true OTUs was better for the shorter V6 region than for the longer V3–V4 regions (Table 5). However, a better estimate of the actual number of OTUs was obtained through analysis of the V3––V4 regions (14–23 observed OTUs vs. 22–35 observed OTUs for *iv*-SC V6P, Table 5). All three de-noising algorithms appear to function similarly well for analysis of the V6 region. For the V3V4P *iv*-SC, PyroTagger and SLP appear better at predicting true OTUs (10 and 8 OTUs, respectively) than AmpliconNoise (4 OTUs). However, it should be noted that several OTUs were missing completely from the community reconstructions performed with SLP and PyroTagger (7 and 3 OTUs, respectively), whereas AmpliconNoise produced the highest number of relevant (i.e., true + miscalled + near-match) OTUs (Table 5). A closer examination of OTUs missing from SLP reconstruction revealed that reads that should comprise these missing OTUs were present in the original quality-screened dataset and that the SLP de-noising algorithm itself had over-clustered these reads into a single read prediction represented by a true sequence (Table S4). This behavior was only observed for SLP de-noising of the V3V4P *iv*-SC, and it performed well for V6P *iv*-SC.

**Figure 3:**
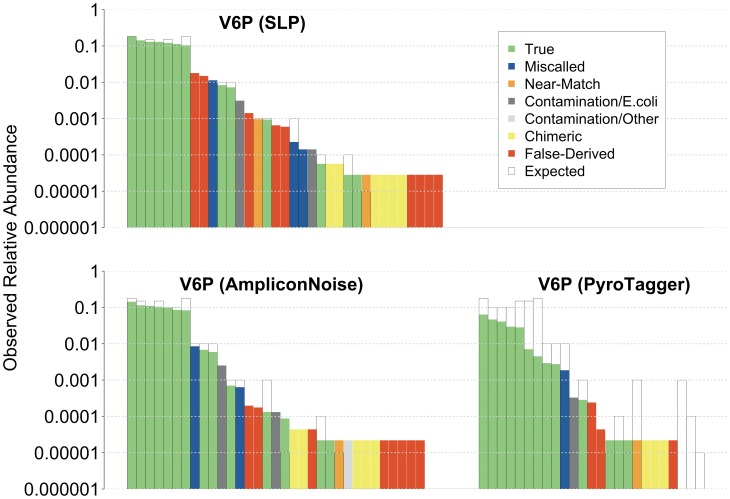
Rank-frequency plots of V6P OTUs generated by SLP, AmpliconNoise, and PyroTagger. Abundances are shown in log scale. True OTUs (green): OTUs with a reference sequence as its representative; Miscalled OTUs (blue): OTUs containing a reference sequence, but not as its representative; False-Derived OTUs (red): OTUs composed entirely of erroneous reads that are not chimeric, contamination, or closely matching a reference sequence not found in any True or Miscalled OTUs; Near-Match OTUs (orange): OTUs containing sequence(s) that closely match a reference sequence not found in any True or Miscalled OTUs; Contamination/*E. coli* (dark gray): OTUs composed of sequences affiliated with *E. coli* (cloning host); Contamination/Other (light gray): OTUs composed of sequences affiliated with potential contaminants; Chimeric OTUs (yellow): OTUs composed of manually identified chimeric sequences; Theoretical (white): expected OTUs.

Rank-frequency plots of OTU types generated from the V6P *iv*-SC (Figure 3) compare observed and expected frequencies for a given clone sequence. Chimeric and false-derived OTUs made up a significant portion of the rare OTUs identified by each de-noising algorithm, and these were indistinguishable from true OTUs at similarly low abundances. False-derived OTUs were observed at >1% relative abundance with SLP, suggesting that even relatively abundant OTUs may be attributable to methodological artifact and that the frequencies of these false-derived OTUs can number as high as 12–13% of the true OTUs from which they are derived (Table S4). The frequencies of false-derived OTUs detected using PyroTagger and AmpliconNoise were notably lower (<0.1%, see Figure 3 and Table S4), but similarly, some of the rare false-derived OTUs had rather high relative abundances to the true OTUs from which they are derived (>27%).

Correlations between observed and expected OTU frequencies were examined using true, miscalled, and near-match (i.e., relevant) OTUs (Table 6). For the relevant OTUs, community structure estimates based on de-noised reads were not significantly different from those based on error-free reads. It should be noted that the community reconstructed using AmpliconNoise was in marginally better agreement with the expected structure for both the V3V4P and V6P *iv*-SCs than that from error-free reads. SLP performed similarly well for the V6P *iv*-SC, but not for V3V4P (Table 6).

**Table 6:** Spearman rank (ρ) and log-log transformed Pearson (*r*) correlation coefficients of true and miscalled OTUs identified by de-noising algorithms with their respective expected frequencies.

Community	De-noising approach	Relevant OTUs	Spearman ρ	Pearson *r*
V3V4P	None (from Table 3)	15	0.941	0.943
	SLP	8	0.752	0.880
	AmpliconNoise	14	0.967	0.977
	PyroTagger	11	0.711	0.827
V6P	*None (from * *Table 3* *)*	*19*	0.929	0.945
	SLP	18	0.929	0.969
	AmpliconNoise	17	0.935	0.969
	PyroTagger	15	0.864	0.928

### Pyrosequencing-Specific Chimera Identification

Unique among the pipelines evaluated, AmpliconNoise explicitly integrated a chimera removal algorithm, Perseus, into its analysis pipeline [19]. Perseus was also applied to de-noised reads from SLP and PyroTagger. Examination of datasets inclusive of chimeric reads revealed that although chimeric reads represent a small portion of the overall *iv*-SC (<1%) (Table 7), they can contribute significantly to overall estimates of OTU richness. Inclusion of chimeric reads increased the number of V3V4P *iv*-SC OTUs reconstructed using both AmpliconNoise and PyroTagger. A close examination of V3V4P *iv*-SC OTUs reconstructed with SLP revealed that 128 of the 345 chimeric sequences in the dataset de-noised using AmpliconNoise were also found in the SLP dataset, but these chimeric reads had been “absorbed” into a true OTU by aggressive clustering in the SLP algorithm. Similarly, 20 of these 345 chimeric reads had been “absorbed” into non-chimeric predicted reads by PyroTagger. Perseus did not identify any chimeric reads in the V6P *iv*-SC, regardless of the de-noising pipeline used. Despite these efforts, several OTUs composed of chimeric reads that had evaded Perseus were manually identified in V3V4P and V6P *iv*-SCs, and they typically comprised ∼15% of the observed OTUs (Table 5).

**Table 7:** Summary statistics for chimera-check of *iv*-SCs.

		Unique De-noised Reads	Replicated De-noised Reads	% of unique reads removed with Perseus (#)	% of replicated reads removed with Perseus (#)	Perseus-Pass OTUs	OTUs Removed by Perseus
V3V4P	SLP	15	36949	0% (0)	0% (0)	14	0
	AmpliconNoise	70	43645	34% (24)	0.8% (345)	23	22
	PyroTagger	20	4475	25% (5)	0.09% (7)	15	5
V6P	SLP	35	35032	0% (0)	0% (0)	35	0
	AmpliconNoise	36	34855	0% (0)	0% (0)	33	0
	PyroTagger	22	10443	0% (0)	0% (0)	22	0

## Discussion

The use of Roche 454 GS FLX next generation sequencing has played an instrumental role in introducing the concept of a *Rare Biosphere*, with this long tail of rare taxa being reported for nearly every community characterized using 454 pyrosequencing [2], [4], [7], [9], [32]–[36]. Although the presence of rare taxa in various environments has been shown using a variety of independent methods [37]–[42], the true frequencies of these taxa, particularly as characterized using pyrosequencing data, remain in question [15], [17]. Moreover, little is known about the accuracy of community structural information derived from the frequency distribution of 16S rRNA gene amplicons within 454 pyrosequencing libraries obtained using the newer Titanium chemistry with longer read lengths.

Overall, our findings show that the 454-Ti sequencing platform provides useful information about microbial community structure since observed and expected frequencies of error-free reads exhibited good correlations (Table 2). Effects of pyrosequencing-specific biases (based on “P” *iv*-SCs) were exceeded by the impact of PCR biases in mixed template samples (“T” and “E” *iv*-SCs). For example, nearly half of the error-free reads in V3V4T originated from a single sequence (1216C) at only 1% relative abundance within the template DNA (Table S2), and the positive PCR bias for this sequence in the V3–V4 regions resulted in a significant skew in recovered community structure information (Table 2 and Table 3). Therefore, the presence of one or a few sequences prone to PCR bias can drastically skew observed relative abundances, but the rank frequency distribution of other sequences appears to be preserved (Table 3). Meanwhile, the V6 *iv*-SCs did not appear to have been subject to significant PCR bias.

Error-containing reads comprised a significant portion of the total reads for all *iv*-SCs. However, this was not due to quality issues with the sequencing process, as the observed proportions are in fact consistent with a high per-base accuracy (>99.5%, Table S1). Therefore, it would have been impossible to systematically isolate the error-containing reads without *a priori* knowledge of the community. To resolve this issue, “de-noising” algorithms that employ clustering techniques were used to assign error-containing sequences to the true sequences from which they arose [17]–[23]. Our findings showed that these approaches occasionally infer the wrong “true” sequence from clusters of mixed error-free and error-containing reads, and invariably produced low-abundance false OTUs that are indistinguishable from real ones. These false OTUs can lead to an overestimation of the total number of OTUs in the *iv*-SCs. In some cases, over-clustering by the de-noising algorithm compensated, albeit incorrectly, for this OTU inflation. Nevertheless, these de-noising algorithms represent a marked improvement over simple, arbitrary quality filters [6], [16] in that they effectively reduce the number of unique error-containing reads that can be mistaken for real sequences.

Although these de-noising pipelines were evaluated in their respective primary publications for the accuracy of recovered richness [17] and relative abundances [19]–[21], this study provides the first independent, explicitly quantitative assessment of their performance using carefully constructed and well quantified *in vitro*-simulated communities. Given that researchers interpreting results from these pipelines inevitably treat them as quantitatively representative of the biological communities, the results presented here provide a useful assessment of information obtained and disseminated using such methodology. A step-by-step comparison between the three de-noising algorithms was unfeasible due to their integrated pipeline design.

The process of clustering sequencing reads into OTUs traditionally involves three distinct steps: quality filtering, alignment, and clustering. The SLP and AmpliconNoise de-noising step constitutes an independent procedure that occurs after quality filtering but before alignment [17], [19]. PyroTagger instead combines de-noising, alignment and clustering into a single, final step [20]. It should be noted that PyroTagger’s authors pointed out that it may not be suitable for 454-Ti data due to supposedly lower read quality, but given that 454-Ti has become the *de facto* technology for amplicon sequencing, we felt that an assessment of the unique approach employed by PyroTagger needed to be included. AmpliconNoise was chosen over alternative flowgram-based clustering algorithms for several reasons: 1) it incorporates a number of significant performance improvements over PyroNoise [21]; 2) its implementation allows it to be run on a computer cluster to speed up analysis; 3) it does not incorporate a greedy/heuristic step and thus has better reproducibility (vs. Qiime Denoiser [23], Figure S1). We note that the two central components of AmpliconNoise, PyroNoise and SeqNoise [19], have recently been re-implemented in Mothur as the Shhh.flows command, which was shown to perform comparably to AmpliconNoise under similar circumstances [18].

Correlations between OTU frequencies calculated from de-noised reads and expected OTU relative abundances were similar to those calculated from error-free reads, indicating that these methods can effectively recover error-containing reads while maintaining approximate community structure. All three de-noising approaches identified similar numbers of OTUs that reflected real *iv*-SC taxa (i.e., true, miscalled and near-known OTUs), but differed in the numbers of false OTUs detected, with PyroTagger outperforming both SLP and AmpliconNoise (Figure 3 and Table 5). However, PyroTagger produced the poorest correlation between observed and expected relative abundances (Table 6) and incorrectly merged reference V3–V4 sequences, indicating a tendency to over-cluster. The stringent quality-based filtering used by PyroTagger also discarded a greater number of raw sequencing reads (data not shown), resulting in the absence of several expected low-abundance taxa from the de-noised dataset (Figure 3 and Table 5).

SLP performed similarly to PyroTagger in predicting species richness within the V3V4P community, but did so by an over-aggressive de-noising procedure that resulted in several real taxa being erroneously grouped into one OTU. This occurred at the de-noising step and was not related to post de-noising clustering procedures (data not shown). Moreover, SLP inferred abundant (>1%) OTUs comprised entirely of error-containing reads in the reconstruction of the V6P *iv*-SC. Compared to SLP, false-derived OTUs were observed at much lower frequencies (<0.1%) for the V6P iv-SC reconstructed using either PyroTagger or AmpliconNoise. Although more computationally intensive, AmpliconNoise models the distribution of pyrosequencing errors at the flowgram level and is able to robustly assign error-containing reads to their parent error-free reads. AmpliconNoise appears to be free from the over-clustering effect observed with both PyroTagger and SLP, and therefore tends to overestimate OTU richness (Table 5). However, it incorrectly identified the highest number of OTU representative sequences with the V3V4P *iv*-SC, which may have ramifications for downstream analyses that rely on precise phylogenetic resolution.

Because AmpliconNoise includes a built-in chimera checker, Perseus, it bypasses the need for multiple sequence alignment (MSA) [43] or reference sequences, as recommended for PyroTagger [20]. For typical pyrosequencing amplicon datasets containing thousands of unique sequences, MSA is impractical, as are the use of reference sequences and *a priori* assumptions about the identity of environmental sequences. The outcome of our analyses shows that AmpliconNoise is the de-noising algorithm least likely to allow chimeric reads to be “absorbed” into read predictions (Table 7), thus affecting abundance estimates. This may partially explain why the correlation between the expected and the observed frequencies of relevant OTUs was highest for the AmpliconNoise pipeline (Table 6).

Rather than using mixtures of genomic DNA preparations, plasmids containing cloned 16S rRNA genes were used for this study. This approach avoided the issues of inter-genomic variations in *rrn* operon copy numbers, intra-genomic variation in *rrn* operon sequences, and quantification inaccuracies due to genome size differences [44], thus allowing greater quantitative accuracy. We limited the richness of the *iv*-SCs to twenty sequences to allow reliable quantification of libraries using both mixed plasmids and PCR products. Given the high proportion of artifactual rare OTUs recovered by all three de-noising pipelines with these relatively simple communities, it is unlikely that a more complex simulated community would have improved their performance. Nineteen of the twenty clones included in the study were from *Cyanobacteria* isolated from similar environments and are therefore comparatively similar in sequence. This resulted in some of the reference sequences being clustered together, even by the most lenient clustering approach (Table 5), but it also exposed PyroTagger’s tendency to over-cluster and mask genuine diversity (Table 5). The inclusion of one *Actinobacteria* clone (1216C) allowed us to explore the effects of primer bias on different phylogenetic groups.

Although we had *a priori* knowledge of the *iv*-SC sequences, we elected not to customize PCR primers to account for known mismatches and performed the experiment using “universal” primers commonly used for microbial community analyses [25], [27], [28], [45]. Thus, our analyses were subject to the same biases common to any study utilizing these common universal primers against environmental DNA. We also avoided using primers with degenerate bases since primer degeneracy can reduce specificity, lead to exhaustion of effective primers as the reaction progresses [31], [46], and impose biases of its own [47]. Recently, an alternative of using a mixture of non-degenerate primers has been proposed [46], which may significantly increase “universality” while avoiding the pitfalls of degenerate primers.

Numerous mechanisms can contribute to PCR bias, including polymerase error [48], formation of chimeric and heteroduplex molecules [49]–[51], and differential amplification efficiency [30], [31], [52]. Our study incorporated many of the wet bench techniques known to be effective toward reducing these biases [30], [31], [48], [49], [52], including low cycle numbers (30 cycles), pooling multiple reactions (3×30 µl), high template concentration (>4 ng of 16S rRNA gene clones), and the use of a proofreading DNA polymerase. Differential primer annealing efficiency provides another mechanism for PCR bias, and although factors such as annealing temperature and primer GC content can influence the outcome of PCR [29], [46], [47], primer mismatch may have the greatest impact for PCR studies of 16S rRNA gene diversity.

The lack of a truly “universal” pair of 16S rRNA gene PCR primers has long been acknowledged [28], [29], [45], [46], [53]. Although some have suggested that the number of taxa recovered is not necessarily linked to the taxonomic specificity (i.e., universality) of a primer set [25], our findings suggest that mispriming is a major, if not the main, factor leading to errors in the observation frequency of taxa within a community (Table S3). Mispriming near the 5′ end of the priming region is thought to have little effect on PCR since extension occurs from the 3′ end [54]. However, it has been reported that 454 Fusion primers containing the 454 adapter sequence at the 5′ end may be more susceptible to the effects of mispriming, resulting in the over-representation of templates that are not misprimed [20]. The adoption of a two-step PCR for amplicon pyrosequencing may ameliorate this issue [55]. Moreover, our findings highlight the complications associated with comparing community structures obtained using different primer sets.

Certain aspects of our experimental protocol may have exacerbated effects of PCR primer mismatch. For example, preferential amplification of perfectly matching template would be expected since the annealing temperature in our PCR protocol started high and decreased with each cycle (see Information S1) rather than starting at a lower temperature [28], [29]. Our modified PCR protocol was chosen because it resulted in an increased DNA yield and thus enabled accurate quantification of PCR amplicons (a prerequisite of pyrosequencing of PCR amplicons). This limitation can be addressed by new instruments that enable small quantities of DNA to be precisely characterized (e.g., Agilent 2100 Bioanalyzer, Agilent Technologies), fractionated (e.g., LabChip XT, Caliper Life Sciences), and quantified (e.g., Kapa Library Quant Kits, Kapa Biosystems). Although these methods were not available for this study, we recommend that they be adopted for the preparation of 16S rRNA gene amplicon libraries for 454-Ti sequencing in addition to adopting PCR conditions such as very low *T_m_* [29] and low (<25) PCR cycles (in conjunction with higher template quantity where possible) [48], [49].

Our results have shown that while de-noising methods for pyrosequencing data need further development, they are an essential processing step for the recovery of usable community structure information. Overall, the largest hurdle to accurate estimation of microbial community structure appears to be PCR bias, which is independent of sequencing technology. Although a variety of measures may be taken to reduce the impact of PCR bias, it cannot be eliminated outright, and our findings highlight the need to better characterize this phenomenon using simulated communities. Another source of error also arises from PCR in the form of chimeric sequences, which are difficult to eliminate. Even though Perseus was able to effectively remove a large portion of chimeric sequences, a small portion of chimeric sequences contributed disproportionately to the number of OTUs observed, especially the infrequent (i.e., rare) OTUs (Figure 3 and Table S4). Therefore, chimeras can significantly inflate OTU estimates, even with short PCR amplicons generated from presumably “immune” 16S regions such as the V6 hypervariable region [17] (Table S4). These realities, combined with the observed prevalence of artifactual rare OTUs (Figure 3), caution against singular interpretations of community structure, especially those that involve within-sample relative OTU frequencies or estimations of *Rare Biosphere* diversity. Instead, the strength of the 454-Ti platform more likely lies in comparative studies and identifying the presence of specific rare taxa. Lastly, our findings highlight the dangers in quickly adopting technological advances without statistically robust validation, given that substantial portions of the *Rare Biosphere* identified using up-to-date de-noising algorithm are still artifacts. The impressively high microbial diversities reported by some past studies [3], [5], [9], [33], [56] based on less developed pyrosequencing quality filters should therefore be re-examined.

## Materials and Methods

### Preparation of 16S rRNA Gene PCR Clones

Twenty bacterial 16S rRNA gene PCR clones were obtained from two previous studies: 19 taken from fresh water habitats in New Zealand [57], and one from Adelie penguin fecal swab samples taken from Antarctica [58]. The primers used to generate initial PCR products (338F/modified 23S30R and EubB/ITSReub) and PCR cloning procedures were as described previously [57], [58]. Briefly, PCR products were gel-purified and cloned using the TOPO TA Cloning Kit (Invitrogen Corp., Carlsbad, CA) following the manufacturer’s instructions. The resulting clones were screened, isolated, and sequenced bi-directionally on an ABI 3730×l DNA Analyzer (Applied Biosystems, Foster City, CA). All 20 plasmids were verified to contain a unique and known insert of the 16S rRNA gene including the V3–V4 and V6 hypervariable regions. All clones except one (1216C: unclassified *Clostridia*) affiliate with members of *Cyanobacteria*.

### Generation of *in vitro*-Simulated Communities and Pyrosequencing

Plasmid preparations were quantified using a NanoDrop ND-1000 UV-Vis spectrophotometer (NanoDrop Technologies, Wilmington, DE) and the QuBit dsDNA HS fluorometric kit (Invitrogen); both methods were repeated in triplicate. Purified plasmid preparations were pooled at known abundances to construct two *in vitro*-simulated communities (*iv*-SCs): uniformly equal (E) and tiered (T) (Table 1). The pooled plasmid DNA sample was treated with Plasmid-Safe ATP-Dependent DNase (EPICENTRE Biotechnologies, Madison, WI) to remove contaminating genomic DNA from cloning hosts (i.e., *E. coli*). PCR amplicon libraries of the V3–V4 (*iv*-SCs: V3V4E & V3V4T) and V6 (*iv*-SCs: V6E & V6T) hypervariable regions were generated using these mixed plasmid communities as templates (454 Fusion PCR primers listed in Table 4). See Information S1 for PCR components and conditions, and quality control for PCR amplicons. An additional set of PCR-neutral *iv*-SCs (P) was constructed using PCR products individually amplified from each plasmid and subsequently pooled in tiered compositions (*iv*-SCs: V3V4P & V6P) after gel extraction and quantification as described in Information S1. The resulting *iv*-SCs were shipped frozen to the J. Craig Venter Institute, where emPCR was performed separately on pooled V3–V4 and V6 *iv*-SCs. The *iv*-SCs were pooled at the following ratios: “T”, 40%; “E”, 20%; and “P”, 40%. The two emPCR libraries were pooled together and sequenced from the A adapter using the Roche GS FLX with Titanium chemistry using one of two regions on a GS FLX Titanium PicoTiterPlate. Original pyrosequencing flowgram files are available from Sequence Read Archive (http://www.ebi.ac.uk/ena/data/view/ERP001633).

### Identification of Error-Free Reads

Read sequences and corresponding quality files were generated using standard Roche software. Reads were compared to the expected amplicon products from V3–V4 and V6 regions of known clone sequences to determine the numbers of error-free reads corresponding to each target. Reads were required to match known sequences exactly over the amplified region, excluding primer sequences. Sequences with a perfect match to the known plasmid insert sequence and spanning the entire V3–V4 or V6 region were used in frequency calculations. In the case of the longer V3–V4 amplicons, sequences were also allowed to terminate prematurely if they were at least 216 nt in length (post primer trim), the minimum needed for each known sequence to be unequivocally identified.

### Sequence Processing and OTU Determination

Prior to workflow-specific quality filtering and de-noising procedures, read sequences and corresponding quality files were generated using standard Roche software. Reads that did not perfectly match the expected primer and MID sequences were discarded. Among the remaining reads, primer and MID sequences were trimmed after reads were separated into individual files by *iv*-SCs.

#### Single-linkage preclustering (SLP) [17]

Reads with one or more ambiguous bases (N, quality score = 0) were removed. Average quality score was then calculated for every remaining read: those with an average quality score of less than 30 were discarded. Reads shorter than a specified length (50 nt) were also discarded. The SLP Perl script was used to assign low-frequency reads to higher frequency reads (http://vamps.mbl.edu/resources/software.php, downloaded in May 2011). Pairwise distances were calculated using Esprit [59]. For pre-clustering, a width of 0.02 was used, and an OTU size of 10 sequences was used for iterative clustering. The resulting datasets were screened for chimeras using Perseus (α = −7.5, β = 0.5) [19]. Esprit was used to calculate pairwise distances for unique sequences, which were then clustered into OTUs using Mothur 1.17.0 [60] at an average neighbor distance of 0.03, as recommended by the SLP authors [17].

#### PyroTagger [20]

Reads were length-trimmed to a specific length (60 nt for V6 amplicons and 216 nt for V3–V4 amplicons) after removal of primer sequences. All remaining reads with ≥3% bases having Q-scores ≤27 were removed from the dataset. PyroTagger, with the pyroclust option, was used to assign quality-filtered reads directly into OTUs without an alignment-based distance calculation step. To do this, sequences were first sorted by abundance and de-replicated. Chimeras were removed using Perseus (α = −7.5, β = 0.5) [19]. Unique reads were then clustered to form OTUs at 97% sequence identity using pyroclust’s default parameters.

#### AmpliconNoise [19]

Raw flowgrams (.sff files) were filtered based on primer and MID sequences match, and the occurrence of the first noisy cycle (i.e., 0.5–0.7 or no signal in all four nucleotide flows). For V6 amplicon reads, flowgrams were truncated at the first noisy cycle, whereas V3–V4 amplicon reads were dropped if the first noisy cycle occurred before cycle 360. The flowgrams were then de-noised using PyroNoise (cluster size = 60, initial cutoff = 0.01), and the resulting sequences were truncated at 400 nt for V3–V4 amplicons and 200 nt for V6 (although no V6 actually exceeded this length). In the final de-noising step, SeqNoise (cluster size = 30, initial cutoff = 0.08) was used. MID and primer sequences were trimmed from the resulting sequence predictions. Chimeras were removed using Perseus (α = −7.5, β = 0.5) [19]. The resulting de-noised, unique reads were aligned using mafft [61], [62], and the alignment was imported into Mothur [60] to construct a pairwise distance matrix using the dist.seqs function, ignoring terminal gaps. Sequences were then clustered into OTUs with an average neighbor clustering distance of 0.03.

## Supporting Information

Figure S1 **Comparison of AmpliconNoise vs. Qiime Denoiser workflows.**
(TIFF)

Table S1 **Summary statistics for 16S rRNA gene amplicon sequence libraries of each** ***iv*** **-SC.**
(DOCX)

Table S2 **Actual relative abundances of each sequence in each** ***iv*** **-SC based on error-free reads.**
(DOCX)

Table S3 **PCR primer mismatches and their impact on the ratio of observed vs. expected frequencies (O:E).** F or R indicates forward or reverse primer, respectively; number designates the position of the mismatch numbered from the 5' end. Observed to Expected Ratios (O:E) were calculated from Table S2.
(DOCX)

Table S4 **Identity of OTUs produced by AmpliconNoise (with Perseus) and SLP from V3V4P and V6P.**
(DOCX)

Information S1 **Additional Material and Methods.**
(DOCX)
