# Supplementary material for: Groundtruthing Next-Gen Sequencing for Microbial Ecology–Biases and Errors in Community Structure Estimates from PCR Amplicon Pyrosequencing
Source: PLoS One. 2012 Sep 6;7(9):e44224. doi: 10.1371/journal.pone.0044224 (PMC3435322; doi:10.1371/journal.pone.0044224)
Supplement: Table S1 — Summary statistics for 16S rRNA gene amplicon sequence libraries of each iv -SC. (DOCX) [file pone.0044224.s002.docx]

Table S1: Summary statistics for 16S rRNA gene amplicon sequence libraries of each *iv*-SC.

| Amplicon Region | Community | Total Reads | Contaminating Reads^*^ | Errant Reads | Errant % | Calculated Per-Base Accuracy |
| --- | --- | --- | --- | --- | --- | --- |
| V3-V4 | V3V4T | 42,721 | 143 | 33,148 | 78% | 99.63% |
|  | V3V4P | 75,447 | 10,266 | 55,394 | 73% | 99.67% |
|  | V3V4E | 31,330 | 99 | 24,938 | 80% | 99.61% |
| V6 | V6T | 71,070 | 6 | 25,036 | 35% | 99.28% |
|  | V6P | 53,654 | 44 | 17,216 | 32% | 99.36% |
|  | V6E | 27,165 | 0 | 10,958 | 40% | 99.14% |

^*^Reads directly attributable to *E. coli* strain used for cloning.
