# Supplementary material for: Groundtruthing Next-Gen Sequencing for Microbial Ecology–Biases and Errors in Community Structure Estimates from PCR Amplicon Pyrosequencing
Source: PLoS One. 2012 Sep 6;7(9):e44224. doi: 10.1371/journal.pone.0044224 (PMC3435322; doi:10.1371/journal.pone.0044224)
Supplement: Table S2 — Actual relative abundances of each sequence in each iv -SC based on error-free reads. (DOCX) [file pone.0044224.s003.docx]

Table S2: Actual relative abundances of each sequence in each *iv*-SC based on error-free reads.

|  |  | Community | | | | | |
| --- | --- | --- | --- | --- | --- | --- | --- |
| Clone | Expected^*^ | V3V4P | V6P | V3V4E | V6E | V3V4T | V6T |
| A32_4-3Okaro10 | 0.18 | 0.30 | 0.22 | 0.0048 | 0.041 | 0.29 | 0.32 |
| A34_SC8-3 | 0.18 | 0.20 | 0.090 | 0.0033 | 0.029 | 0.062 | 0.079 |
| A09_SC7-1 | 0.15 | 0.094 | 0.14 | 0.0011 | 0.032 | 0.017 | 0.072 |
| A16_LMM1-5 | 0.15 | 0.12 | 0.14 | 0.0046 | 0.083 | 0.067 | 0.19 |
| A05_SC1-5 | 0.10 | 0.047 | 0.11 | **0** | 0.024 | 0.0048 | 0.057 |
| A38_3-9 | 0.10 | 0.13 | 0.14 | 0.0063 | 0.11 | 0.045 | 0.17 |
| A35_23-7 | 0.05 | 0.0094 | 0.059 | 0.0017 | 0.084 | 0.0044 | 0.055 |
| A36_30-1 | 0.05 | 0.070 | 0.070 | 0.0063 | 0.089 | 0.033 | 0.056 |
| A10_19-3 | 0.01 | 0.0085 | 0.0095 | 0.0089 | 0.036 | 0.0076 | 0.0047 |
| A14_16-1 | 0.01 | **0** | 0.0039 | **0** | 0.015 | **0** | 0.0016 |
| J02_0239_1216C | 0.01 | 0.023 | 0.0098 | 0.83 | 0.0014 | 0.47 | 0.00020 |
| A08_SC5-2 | 0.001 | 0.0031 | 0.00003 | 0.0080 | 0.00061 | 0.00076 | 0.00003 |
| A11_29-2 | 0.001 | **0** | 0.00021 | **0** | **0** | **0** | **0** |
| A31_Forsyth-N6 | 0.001 | 0.00018 | 0.0011 | 0.00043 | 0.049 | **0** | 0.00075 |
| A04_Wahhi-22 | 0.0001 | 0.00037 | 0.00006 | 0.0078 | 0.057 | **0** | 0.00008 |
| A06_SC4-1 | 0.0001 | 0.00018 | 0.00006 | 0.0028 | 0.083 | **0** | 0.00008 |
| A12_3-1 | 0.0001 | 0.00055 | 0.00018 | 0.11 | 0.099 | 0.00045 | 0.00015 |
| A13_6-1 | 0.00001 | **0** | 0.00003 | 0.00065 | 0.016 | **0** | **0** |
| A15_EF222209 | 0.00001 | **0** | 0.00009 | 0.0046 | 0.14 | **0** | **0** |
| A17_LMM1-24 | 0.00001 | **0** | **0** | 0.0026 | 0.0079 | **0** | **0** |

^*^For P & T samples.
