# Supplementary material for: Groundtruthing Next-Gen Sequencing for Microbial Ecology–Biases and Errors in Community Structure Estimates from PCR Amplicon Pyrosequencing
Source: PLoS One. 2012 Sep 6;7(9):e44224. doi: 10.1371/journal.pone.0044224 (PMC3435322; doi:10.1371/journal.pone.0044224)
Supplement: Table S3 — PCR primer mismatches and their impact on the ratio of observed vs. expected frequencies (O:E). F or R indicates forward or reverse primer, respectively; number designates the position of the mismatch numbered from the 5' end. Observed to Expected Ratios (O:E) were calculated from Table S2. (DOCX) [file pone.0044224.s004.docx]

Table S3: PCR primer mismatches and their impact on the ratio of observed vs. expected frequencies (O:E). F or R indicates forward or reverse primer, respectively; number designates the position of the mismatch numbered from the 5' end. Observed to Expected Ratios (O:E) were calculated from Table S2.

| Clone | V3V4 Primer Match | V3V4E O:E | V3V4T O:E | V6 Primer Match | V6E O:E | V6T O:E |
| --- | --- | --- | --- | --- | --- | --- |
| Waahi-22 | R8: C🡪A; R9: A🡪G | 0.156 | 0 | Perfect | 1.145 | 0.750 |
| SC1-5 | R8: C🡪A; R9: A🡪G | 0 | 0.048 | Perfect | 0.477 | 0.570 |
| SC4-1 | R8: C🡪T; R9: A🡪G | 0.056 | 0 | Perfect | 1.656 | 0.750 |
| SC5-2 | R8: C🡪T; R9: A🡪G | 0.161 | 0.757 | F9: G🡪A | 0.012 | 0.025 |
| SC7-1 | R8: C🡪T; R9: A🡪G | 0.022 | 0.110 | Perfect | 0.644 | 0.483 |
| 19-3 | F: Unknown^*^ R8: C🡪A; R9: A🡪G | 0.178 | 0.757 | Perfect | 0.728 | 0.465 |
| 29-2 | R8: C🡪T; R9: A🡪G | 0 | 0 | F2: A🡪G | 0 | 0 |
| 3-1 | R9: A🡪G | 2.100 | 4.540 | Perfect | 1.974 | 1.500 |
| 6-1 | F: Unknown^*^ R8: C🡪A; R9: A🡪G | 0.0130 | 0 | Perfect | 0.327 | 0 |
| 16-1 | F1: A🡪T; F2: C🡪A F4: C🡪T; R8: C🡪T; R9: A🡪G | 0 | 0 | Perfect | 0.302 | 0.160 |
| EF222209 | R8: C🡪T; R9: A🡪G | 0.091 | 0 | Perfect | 2.841 | 0 |
| LMM1-5 | R8: C🡪A; R9: A🡪G | 0.091 | 0.444 | Perfect | 1.652 | 1.242 |
| LMM1-24 | R8: C🡪A; R9: A🡪G | 0.052 | 0 | Perfect | 0.159 | 0 |
| Forsyth-N6 | R8: C🡪T; R9: A🡪G | 0.009 | 0 | Perfect | 0.981 | 0.750 |
| 4-3Okaro10 | R8: C🡪T; R9: A🡪G | 0.095 | 1.617 | Perfect | 0.824 | 1.788 |
| SC8-3 | R8: C🡪T; R9: A🡪G | 0.065 | 0.343 | Perfect | 0.589 | 0.437 |
| 23-7 | R8: C🡪T; R9: A🡪G | 0.035 | 0.088 | Perfect | 1.686 | 1.091 |
| 30-1 | R8: C🡪T; R9: A🡪G | 0.126 | 0.651 | Perfect | 1.787 | 1.128 |
| 3-9 | R8: C🡪T; R9: A🡪G | 0.126 | 0.454 | Perfect | 2.190 | 1.655 |
| 1216C | Perfect | 16.623 | 46.814 | R6: G🡪A | 0.028 | 0.020 |

^*^5’ end sequence of V3-V4 hypervariable region could not be obtained using Sanger sequencing despite multiple attempts using several primers.
