# Supplementary material for: Groundtruthing Next-Gen Sequencing for Microbial Ecology–Biases and Errors in Community Structure Estimates from PCR Amplicon Pyrosequencing
Source: PLoS One. 2012 Sep 6;7(9):e44224. doi: 10.1371/journal.pone.0044224 (PMC3435322; doi:10.1371/journal.pone.0044224)
Supplement: Table S4 — Identity of OTUs produced by AmpliconNoise (with Perseus) and SLP from V3V4P and V6P. (DOCX) [file pone.0044224.s005.docx]

Table S4: Identity of OTUs produced by AmpliconNoise (with Perseus) and SLP from V3V4P and V6P.

| AmpliconNoise V3V4P | | | | | |
| --- | --- | --- | --- | --- | --- |
| OTU | Representative Read | | Copy | Classification | Description |
| 2 | F3TO1RL01AXUCQ | | 17251 | Contamination-*E. coli* | *E. coli* |
| 14 | F3TO1RL01A4UUI | | 6028 | True^*^ | SC8-3 (includes 23-7) |
| 13 | F3TO1RL01BMY44 | | 5113 | Miscalled^*^ | 3-1 (includes 3-9) |
| 11 | F3TO1RL01EWBHX | | 3980 | True | 4-3Okaro10 |
| 21 | F3TO1RL01CGRIW | | 3094 | Miscalled | LMM1-5 |
| 9 | F3TO1RL01AMU6Q | | 2909 | Miscalled | SC7-1 |
| 18 | F3TO1RL01D9AHG | | 2890 | Miscalled | SC1-5 |
| 16 | F3TO1RL01C8AQZ | | 1087 | Miscalled | 30-1 |
| 3 | F3TO1RL01DPHY1 | | 578 | True | 1216C |
| 8 | F3TO1RL01DE7HG | | 159 | Miscalled | 19-3 |
| 17 | F3TO1RL01AH9IY | | 81 | Near-Match | 99% identical to 16-1, which isn’t found in any other OTU |
| 6 | F3TO1RL01BCK2W | | 61 | Contamination-Other | α-Proteobacterium known to associate with Cyanobacteria |
| 22 | F3TO1RL01BV3L0 | | 43 | True | SC5-2 |
| 15 | F3TO1RL01DTUD6 | | 12 | Near-Match | Forsyth-N6 |
| 20 | F3TO1RL01BIVJX | | 6 | Miscalled | Wahhi-22 |
| 1 | F3TO1RL01CLBB1 | | 1 | Chimera | *E. coli* and 4-3Okaro10 |
| 4 | F3TO1RL01D56QM | | 1 | Contamination-Other | α-Proteobacterium (Agrobacterium) |
| 5 | F3TO1RL01BY4CO | | 1 | Contamination-Other | α-Proteobacterium (Caulobacter) |
| 7 | F3TO1RL01AT3B4 | | 1 | Chimera | α-Proteobacterium known to associate with Cyanobacteria and 3-1 |
| 10 | F3TO1RL01A2RJ0 | | 1 | Near-Match | 99% identical to LMM1-24, which isn’t found in any other OTU |
| 12 | F3TO1RL01DOEXY | | 1 | Chimera | *E. coli* and SC8-3 |
| 19 | F3TO1RL01CGNBI | | 1 | Chimera | *E. coli* and unresolved *iv-SC* member |
| 23 | F3TO1RL01BHADV | | 1 | Contamination-Other | Actinobacteria |
| SLP V3V4P | | | | | |
| OTU # | | Representative Read | Copy # | Classification | Description |
| 3 | | F3TO1RL01AT3B4 | 19465 | Contamination - *E.coli* | The SLP prediction perfectly matches E.coli but also map back to complete sequences of SC1-5, SC4-1, SC7-1, 19-3, LMM1-5, 4-3Okaro10, SC8-3,, 30-1, 3-9, and fragments of 23-7, 3-1, and SC5-2 |
| 9 | | F3TO1RL01DBZ5A | 15741 | True | 4-3Okaro10  The SLP prediction also maps to complete SC1-5, SC5-2, SC7-1, LMM1-5, SC8-3, 23-7, 30-1, 3-9 and fragments of 3-1 and Forsyth-N6 |
| 13 | | F3TO1RL01DNWKX | 751 | True | LMM1-5 |
| 4 | | F3TO1RL01CCAXC | 419 | True | 1216C |
| 10 | | F3TO1RL01BQLR3 | 273 | True | 3-9 |
| 11 | | F3TO1RL01AE50Q | 196 | True / ambiguous | SC8-3 SLP predictions are too short to resolve properly, but all reads in SLP prediction map back to SC8-3 |
| 8 | | F3TO1RL01A5GLE | 81 | True | 19-3 |
| 7 | | F3TO1RL01BOFCK | 12 | Contamination-Other | α-Proteobacterium known to associate with Cyanobacteria |
| 12 | | F3TO1RL01CN3L7 | 5 | True | Wahhi-22 |
| 1 | | F3TO1RL01BHCQY | 2 | Contamination-Other | *Enterococcus faecalis* 62 |
| 2 | | F3TO1RL01CLZ77 | 1 | True / Ambiguous | Read is too short to resolve |
| 5 | | F3TO1RL01BTBH0 | 1 | Contamination-Other | α-Proteobacterium - Methylotroph |
| 6 | | F3TO1RL01BY4CO | 1 | Contamination-Other | α-Proteobacterium known to associate with Cyanobacteria |
| 14 | | F3TO1RL01BHADV | 1 | Contamination-Other | Actinobacteria |
| PyroTagger V3V4P | | | | | |
| OTU # | | Representative Read | Copy # | Classification | Description |
| 2 | | F3TO1RL01CFVID | 1328 | Contamination-*E. coli* | *E. coli* |
| 9 | | F3TO1RL01APFOM | 1102 | True | 3-9 (includes 3-1) |
| 11 | | F3TO1RL01AJBNO | 1003 | True | SC8-3 (includes SC4-1 and 23-7) |
| 8 | | F3TO1RL01EFP5X | 484 | True | 4-3Okaro10 |
| 10 | | F3TO1RL01BRO99 | 226 | True | 30-1 |
| 3 | | F3TO1RL01DPHY1 | 104 | True | 1216C |
| 6 | | F3TO1RL01C9S89 | 100 | True | SC7-1 |
| 14 | | F3TO1RL01CJL13 | 52 | True | LMM1-5 |
| 12 | | F3TO1RL01CA4PJ | 32 | Near-match | 99% identical to SC1-5 which isn't found in any other OTU |
| 5 | | F3TO1RL01B14QV | 23 | True | 19-3 |
| 4 | | F3TO1RL01BR6PL | 4 | Contamination-Other | α-Proteobacterium known to associate with Cyanobacteria |
| 7 | | F3TO1RL01AL7EY | 4 | Chimera | *E. coli* and 4-3Okaro10 |
| 1 | | F3TO1RL01BUYFI | 2 | Chimera | *E. coli* and unresolved *iv-SC* member |
| 13 | | F3TO1RL01AKAZB | 2 | True | Wahhi-22 |
| 15 | | F3TO1RL01A01RW | 2 | True | Sc5-2 |
| AmpliconNoise V6P | | | | | |
| 13 | | F3TO1RL01EGO1E | 6544 | True | 4-3Okaro10 |
| 23 | | F3TO1RL01CQM5J | 5202 | True | LMM1-5 |
| 3 | | F3TO1RL01CA0M4 | 5017 | True | 3-1 and 3-9 |
| 25 | | F3TO1RL01A2GUJ | 4788 | True | SC7-1 |
| 8 | | F3TO1RL01AF2KK | 4424 | True | 30-1, 23-7 and SC4-1 |
| 6 | | F3TO1RL01AHFL0 | 3893 | True | SC1-5 |
| 9 | | F3TO1RL01DZRUW | 3790 | True | SC8-3 |
| 10 | | F3TO1RL01BTLSL | 388 | Miscalled | 16-1 |
| 1 | | F3TO1RL01AUK4B | 311 | True | 1216C |
| 26 | | F3TO1RL01CIGG2 | 271 | True | 19-3 |
| 32 | | F3TO1RL01ETEVT | 115 | Contamination-*E. coli* | *E. coli* |
| 5 | | F3TO1RL01AIVK5 | 32 | True | ForsythN-6 |
| 21 | | F3TO1RL01A5EE1 | 29 | Miscalled | SC5-2 |
| 15 | | F3TO1RL01E3WOD | 9 | False | Derived from 1216C |
| 19 | | F3TO1RL01ALGSF | 8 | False | Derived from SC5-2 |
| 29 | | F3TO1RL01E4M5O | 6 | True | 29-2 |
| 31 | | F3TO1RL01DV2WI | 6 | Contamination-*E. coli* | *E. coli* |
| 17 | | F3TO1RL01CBAFV | 4 | True | EF222209 |
| 2 | | F3TO1RL01EDJMU | 2 | Chimera | *E. coli* and unresolved *iv-SC* member |
| 7 | | F3TO1RL01DDIPR | 2 | Chimera | *E. coli* and unresolved *iv-SC* member |
| 27 | | F3TO1RL01CVF3M | 2 | False | Derived from 19-3 |
| 4 | | F3TO1RL01DBJFZ | 1 | True | 6-1 |
| 11 | | F3TO1RL01B1MRM | 1 | Chimera | *E. coli* and unresolved *iv-SC* member |
| 12 | | F3TO1RL01CTOUW | 1 | Near Match | 98% identical to LMM1-24, which isn’t found in any other OTU |
| 14 | | F3TO1RL01ENRY2 | 1 | False | Derived from 4-3Okaro10 |
| 16 | | F3TO1RL01BWFJ4 | 1 | False | Derived from EF222209 |
| 18 | | F3TO1RL01ANX84 | 1 | False | Derived from Wahhi-22 |
| 20 | | F3TO1RL01D44QL | 1 | True | Wahhi-22 |
| 22 | | F3TO1RL01DVOLS | 1 | Chimera | *E. coli* and LMM1-5 |
| 24 | | F3TO1RL01BJYCE | 1 | Chimera | *E. coli* and SC7-1 |
| 28 | | F3TO1RL01C0IZI | 1 | False | Derived from unresolved *iv-SC* member |
| 30 | | F3TO1RL01DZJNR | 1 | Contamination-Other | *Verrucomicrobia* |
| 33 | | F3TO1RL01CQ63V | 1 | False | Derived from 19-1 |
| SLP V6P | | | | | |
| OTU # | | Representative Read | Copy # | Classification | Description |
| 16 | | F3TO1RL01D35BZ | 6506 | True | 4-3Okaro10 |
| 3 | | F3TO1RL01EDG7P | 5029 | True^*^ | 3-9 (includes 3-1) |
| 26 | | F3TO1RL01E3P7J | 4583 | True | LMM 1-5 |
| 10 | | F3TO1RL01C957C | 4562 | True^*^ | 30-1 (includes 23-7) |
| 28 | | F3TO1RL01AEZSL | 4267 | True | SC7-1 |
| 7 | | F3TO1RL01CJ6W7 | 3976 | True | SC1-5 |
| 12 | | F3TO1RL01CR64E | 3696 | True | SC8-3 |
| 20 | | F3TO1RL01DLOQ0 | 632 | False | Derived from LMM1-5 |
| 22 | | F3TO1RL01BPZ8J | 526 | False | Derived from SC7-1 |
| 13 | | F3TO1RL01CM0UM | 400 | Miscalled | 16-1 |
| 1 | | F3TO1RL01DYRUB | 294 | True | 1216C |
| 30 | | F3TO1RL01B4OYN | 255 | True | 19-3 |
| 33 | | F3TO1RL01BKXCC | 110 | Contamination-*E. coli* | *E. coli* |
| 21 | | F3TO1RL01EOYWD | 50 | False | Derived from SC7-1 |
| 24 | | F3TO1RL01AV8W6 | 34 | Near Match | Similar to SC5-2 |
| 6 | | F3TO1RL01AIVK5 | 33 | True | ForsythN-6 |
| 18 | | F3TO1RL01B6WEB | 23 | False | Derived from 1216C |
| 4 | | F3TO1RL01C2FC0 | 21 | False | Derived from 3-9 |
| 31 | | F3TO1RL01AUJV3 | 8 | Miscalled | 29-2 |
| 19 | | F3TO1RL01A7LJR | 5 | Miscalled | EF222209 |
| 32 | | F3TO1RL01AKETG | 5 | Contamination-*E. coli* | *E. coli* |
| 2 | | F3TO1RL01EDJMU | 2 | Chimera | *E. coli* and unresolved *iv-SC* member |
| 9 | | F3TO1RL01EJ4WY | 2 | Chimera | *E. coli* and unresolved *iv-SC* member |
| 23 | | F3TO1RL01ANX84 | 2 | True | Wahhi-22 |
| 5 | | F3TO1RL01DBJFZ | 1 | True | 6-1 |
| 8 | | F3TO1RL01DWCLH | 1 | False | Derived from SC1-5 |
| 11 | | F3TO1RL01BK7JK | 1 | True | SC4-1 |
| 14 | | F3TO1RL01B1MRM | 1 | Chimera | *E. coli* and unresolved *iv-SC* member |
| 15 | | F3TO1RL01CTOUW | 1 | Near Match | 98% identical to LMM1-24, which isn’t found in any other OTU |
| 17 | | F3TO1RL01ENRY2 | 1 | False | Derived from 4-3Okaro10 |
| 25 | | F3TO1RL01DVOLS | 1 | Chimera | *E. coli* and LMM1-5 |
| 27 | | F3TO1RL01BJYCE | 1 | Chimera | *E. coli* and SC7-1 |
| 29 | | F3TO1RL01D1HPU | 1 | False | Derived from SC7-1 |
| 34 | | F3TO1RL01CQ63V | 1 | False | Derived from 19-1 |
| 35 | | F3TO1RL01BQ0IE | 1 | Chimera | *E. coli* and 4-3Okaro10 |
| PyroTagger V6P | | | | | |
| OTU # | | Representative Read | Copy # | Classification | Description |
| 11 | | F3TO1RL01C5UW3 | 2903 | True | 4-3Okaro10 |
| 2 | | F3TO1RL01AY06C | 2122 | True^*^ | 3-9 (includes 3-1) |
| 6 | | F3TO1RL01AFBR0 | 1863 | True^*^ | 30-1 (includes 23-7) |
| 5 | | F3TO1RL01D1QS6 | 1347 | True | SC1-5 |
| 17 | | F3TO1RL01B5NGH | 1288 | True | LMM1-5 |
| 18 | | F3TO1RL01CMQV2 | 321 | True | SC7-1 |
| 8 | | F3TO1RL01DS2YP | 206 | True | SC8-3 |
| 1 | | F3TO1RL01DM38N | 134 | True | 1216C |
| 19 | | F3TO1RL01B4OYN | 125 | True | 19-3 |
| 9 | | F3TO1RL01B7X57 | 85 | Miscalled | 16-1 |
| 20 | | F3TO1RL01D685M | 15 | Contamination-*E. coli* | *E. coli* |
| 4 | | F3TO1RL01BRHR3 | 13 | True | Forsyth-N6 |
| 15 | | F3TO1RL01CPXR1 | 11 | False | Derived from LMM1-5 |
| 16 | | F3TO1RL01EW7MO | 2 | False | Derived from SC7-1 |
| 3 | | F3TO1RL01DBJFZ | 1 | True | 6-1 |
| 7 | | F3TO1RL01BK7JK | 1 | True | SC4-1 |
| 10 | | F3TO1RL01B1MRM | 1 | Chimera | *E. coli* and one of several known OTUs |
| 12 | | F3TO1RL01BWFJ4 | 1 | False | Derived from EF222209 |
| 13 | | F3TO1RL01CBAFV | 1 | True | EF222209 |
| 14 | | F3TO1RL01CXA09 | 1 | Near-Match | Similar to SC5-2, which isn’t found in any other OTU |
| 21 | | F3TO1RL01CQ63V | 1 | Chimera | *E. coli* and 23-7 |
| 22 | | F3TO1RL01BQ0IE | 1 | Chimera | *E. coli* and 4-3Okaro10 |

^*^Reference sequences also clustered as one OTU.
