# Supplementary material for: Groundtruthing Next-Gen Sequencing for Microbial Ecology–Biases and Errors in Community Structure Estimates from PCR Amplicon Pyrosequencing
Source: PLoS One. 2012 Sep 6;7(9):e44224. doi: 10.1371/journal.pone.0044224 (PMC3435322; doi:10.1371/journal.pone.0044224)
Supplement: Information S1 — Additional Material and Methods. (DOCX) [file pone.0044224.s006.docx]

SUPPLEMENTARY INFORMATION

*PCR Components and Conditions, and Quality Control for PCR Amplicons*

Triplicate PCRs of 30 µl were performed with 4 to 10 ng of template DNA per tube. The PCR components included 1X PrimeSTAR buffer (final [Mg2+] = 5 mM), 0.2 mM dNTPs, 1.5 unit of PrimeSTAR HS proofreading DNA polymerase (Takara Bio Inc., Shiga, Japan), 400 nM of each primer, and de-ionized water. PCR master mixes were treated with 1 µg/ml ethidium monoazide to deactivate contaminating DNA in PCR reagents [101]. All PCR runs were performed on a Bio-Rad DNA Engine (PTC-200, Bio-Rad Laboratories Inc, Hercules, California) under the following conditions: 94ºC for 2 minutes, followed by 30 cycles of 94ºC for 20 seconds, 55ºC for 10 seconds (-0.2ºC per cycle) for the V3-V4 region or 60ºC for 10 seconds (-0.3ºC per cycle) for V6 region, 72ºC for 20 seconds, and a final extension at 72ºC for 3 minutes. Replicate PCR tubes were pooled and visualized on 2% TAE agarose gel. Amplicon bands were excised and then purified using a QuickClean 5M Gel Extraction kit (GenScript Corporation, Piscataway, New Jersey).

*High-throughput sequencing results in a predictable number of error-containing reads*

Six *in vitro*-simulated communities (*iv*-SCs) were sequenced and reads were generated using standard Roche software. Between 25,000 and 80,000 reads were generated per community (Table S1). Between 30-40% of individual V6-spanning reads contained at least one error and between 70-80% of V3V4-spanning reads contained at least one error. These error rates were used to calculate estimated per-base accuracy rates (Errant%=1-(per-base accuracy)^Length^) where the length for the V6 region is 60 nucleotides and the length of the V3V4 region is 405 nucleotides. As shown in Table S1, the estimated per-base accuracy of this sequencing run exceeded 99.5% for each of the six *iv*-SCs.

*Calculation of statistical significance*

The significance of the difference in correlation between expected and observed community abundances was tested using the r.test() function in the psych package of R (Revelle, W. [2010] psych: Procedures for Personality and Psychological Research Northwestern University, Evanston, http://personality-project.org/r/psych.manual.pdf, 1.0-93). This procedure takes into account sample sizes.

REFERENCES

101. Rueckert A, Morgan HW (2007) Removal of contaminating DNA from polymerase chain reaction using ethidium monoazide. J Microbiol Methods 68: 596–600. doi:10.1016/j.mimet.2006.11.006.
